# ToxEvaluator: an integrated computational platform to aid the interpretation of toxicology study-related findings

**DOI:** 10.1093/database/baw062

**Published:** 2016-05-08

**Authors:** D. Pelletier, T. C. Wiegers, A. Enayetallah, C. Kibbey, M. Gosink, P. Koza-Taylor, C. J. Mattingly, M. Lawton

**Affiliations:** ^1^Pfizer Worldwide Research & Development, Groton, CT 06340; ^2^Department of Biological Sciences, North Carolina State University, Raleigh, NC 27695; ^3^Biogen, Cambridge, MA 02142, USA

## Abstract

Attempts are frequently made to investigate adverse findings from preclinical toxicology studies in order to better understand underlying toxicity mechanisms. These efforts often begin with limited information, including a description of the adverse finding, knowledge of the structure of the chemical associated with its cause and the intended pharmacological target. ToxEvaluator was developed jointly by Pfizer and the Comparative Toxicogenomics Database (http://ctdbase.org) team at North Carolina State University as an *in silico* platform to facilitate interpretation of toxicity findings in light of prior knowledge. Through the integration of a diverse set of *in silico* tools that leverage a number of public and proprietary databases, ToxEvaluator streamlines the process of aggregating and interrogating diverse sources of information. The user enters compound and target identifiers, and selects adverse event descriptors from a safety lexicon and mapped MeSH disease terms. ToxEvaluator provides a summary report with multiple distinct areas organized according to what target or structural aspects have been linked to the adverse finding, including primary pharmacology, structurally similar proprietary compounds, structurally similar public domain compounds, predicted secondary (i.e. off-target) pharmacology and known secondary pharmacology. Similar proprietary compounds and their associated *in vivo* toxicity findings are reported, along with a link to relevant supporting documents. For similar public domain compounds and interacting targets, ToxEvaluator integrates relationships curated in Comparative Toxicogenomics Database, returning all direct and inferred linkages between them. As an example of its utility, we demonstrate how ToxEvaluator rapidly identified direct (primary pharmacology) and indirect (secondary pharmacology) linkages between cerivastatin and myopathy.

## Introduction

During the drug development process, adverse findings from preclinical toxicology studies can raise concerns about similar findings in subsequent human clinical trials. When the adverse findings are serious enough relative to the intended therapeutic indication, development of the molecule may be temporarily or even permanently halted, reducing the availability of new medicines for patients. Depending on a number of independent factors, including the indication and the nature of the finding, an *ad hoc* multidisciplinary team of scientists may be created to further characterize the adverse finding. This ‘issue-management team’ is usually focused on developing an understanding of the underlying molecular mechanism(s) leading to the toxicity, as well as determining the relevance of the adverse finding to human health. The human-relevance point can be critical for continued development of the molecule, as the lack of a direct translation of a preclinical observation has been demonstrated for a number of both marketed and investigational therapeutics (1, 2).

The first step of an investigative team usually involves a period of comprehensive hypothesis generation to explore possible underlying mechanisms. The purpose of this idea-generation stage is to identify the most plausible, testable hypotheses so that resources can be rapidly deployed for follow-up experiments with the most informative outcomes. With a typically limited window of opportunity to derive these hypotheses, efficiency at this stage of work can be critical to the success of the effort.

ToxEvaluator was developed as an *in silico* platform to facilitate rapid and comprehensive hypothesis generation for toxicity findings. Created with a user-friendly interface, ToxEvaluator was designed to integrate the information an investigative toxicologist would typically gather at the start of such a project, and to execute many of the time-consuming analyses needed to explain a toxicity finding. As ToxEvaluator is a proprietary application and therefore not publicly available, the aim of this article is to describe its design and functionality with the intent to inform the development of related applications.

## Design and development

### Proprietary databases and tools

ToxEvaluator was designed to leverage and integrate a number of existing proprietary and publicly available tools and databases that are ideally used in interrogation workflows by investigative toxicologists. Proprietary resources include databases of *in vitro* pharmacological activity, *in vivo* preclinical toxicology study findings (referred to at Pfizer as *in vivo* Data Environment—or IVDE) and compound structure libraries ( > 4 million compounds). The IVDE database includes only study findings that were attributed to the compound, and utilizes an in-house developed safety lexicon of toxicology and pathology terms for describing the findings (see supplementary material).

Proprietary (i.e. developed by Pfizer) *in silico* tools integrated in ToxEvaluator function to predict unintended pharmacology (Polypharmacology), compute compound similarity and link intended pharmacological targets with toxicology-related gene information (ToxReporter). The Polypharmacology tool consists of a collection of 771 individual classification models, one for each target incorporated in the tool. The individual classification models were trained using a combination of internal screening data and bioassay data from external sources including the GVK-Bio GoSTAR database (3) and ChEMBL (4). Similar to other described polypharmacology models (5), this tool enables querying by compound identifier/structure and the return of probabilistic predictions of possible targets with which the compound may interact.

A compound similarity tool is integrated into ToxEvaluator to identify other compounds of potential interest based on structural similarities to the compound being evaluated. The tool utilizes structure-encoding molecular fingerprint descriptors (6) and a Tanimoto similarity algorithm (7) to generate a similarity score within the context of user-defined thresholds. In this way, users may uncover structural analogs from compound libraries and link the similar compounds to reports related to the adverse event being investigated.

The ToxReporter tool links target (gene) information to the Online Mendelian Inheritance in Man (OMIM) (8) via Gene database (9). Links between OMIM entries and toxicity categories are inferred through the MeSH categories of the PubMed articles cited in the OMIM entries. ToxReporter links to the Mouse Genome Informatics (MGI) (10) genetic databases via phenotype information for mouse gene mutations. Finally, ToxReporter incorporates tissue expression data, Gene Ontology data (11) and MeSH-based (12) literature mining functionality to identify and flag sources of information linking a given target with selected organ toxicity terms.

### Comparative Toxicogenomics Database

The Comparative Toxicogenomics Database (CTD) (13) is a publicly available resource that provides compound–gene, compound–disease, and gene–disease interactions and relationships that are manually curated from the scientific literature by professional biocurators. In 2012, CTD’s content was expanded as a result of a collaboration between Pfizer and CTD to text mine and manually curate a collection of over 88 000 articles (14). CTD content has been integrated in ToxEvaluator using Web services specifically developed by North Carolina State University for Pfizer to leverage these curated relationships. The interactions and relationships in CTD enable direct integration with the gene and compound centric output from Pfizer’s proprietary tools. CTD annotates many of the interactions and relationships according to whether there is a direct relationship between the gene–disease and compound–disease pairs [denoted as Marker or Mechanism (M) or Therapeutic (T)], or an inferred relationship between them based on a statistical enrichment of co-interacting third partners (15). ToxEvaluator incorporates all three of these annotation types into its output.

The availability of curated chemical–gene and chemical–disease interactions in CTD allows CTD’s MeSH-based chemical library to be searched for similar compounds that can be linked to the adverse finding. This was accomplished by downloading the CTD chemical library and transforming the structural descriptors for each chemical in Simplified Molecular Input Line Entry System format (16) into the molecular fingerprint descriptors usable by the chemical structural similarity tool integrated in ToxEvaluator. Compounds identified as being similar within the context of user-specified thresholds are readily utilized in chemical–disease queries based on the MeSH chemical identifier that was retained with the structural information in the initial library download.

## ToxEvaluator input

### Ease of use

One important goal of the tool was to improve access to mechanism-based toxicity data for a broad range of users. As such, the query interface was purposefully designed to allow entry of information commonly used for toxicity investigations such as the compound identifier, the target identifier and a description of the adverse finding. By focusing on these three areas and incorporating features such as autocomplete and default similarity thresholds, ToxEvaluator simplifies the input process and facilitates access to integrated results.

### Compound identifier

Input of a compound identifier is critical as a number of workflows depend on the identifier directly or the associated chemical structure. Acceptable compound identifiers include proprietary Pfizer identifiers (e.g. PF-1234567) and common names. Workflows using these identifiers include *in vitro* assay data look-up for secondary pharmacology targets (identifier driven), *in silico* prediction of secondary pharmacology targets (structure driven), and identification of structurally similar compounds using internal (proprietary) and external (CTD) data sets (structure driven). Auto-complete functionality assists the user with input by suggesting compound identifiers in a drop-down box as the initial characters are being entered.

### Target identifier

ToxEvaluator also accepts a primary drug target as input. The majority of drug discovery projects attempts to develop a novel molecule that specifically and potently interacts with an individual, endogenous, target molecule. As the target molecule is typically a protein that is encoded by a gene, a HUGO gene identifier (17) is required for input. A target ID is used by ToxEvaluator to identify links between the target itself and the finding being investigated. An auto-complete functionality assists the user with target input.

### Description of adverse finding

The third piece of required input is a description of the adverse finding. This is critical as the primary function of ToxEvaluator is to find relationships between molecules, targets and the finding of interest. In order for ToxEvaluator to identify these relationships in proprietary and public databases that use different structured vocabularies/ontologies (e.g. CTD—MeSH, IVDE—safety lexicon), a master file of mapped terms was created (see supplementary material). As the adverse finding is often some form of organ or tissue toxicity, MeSH and safety lexicon terms were assigned in the mapping process to organ/tissue terms common to both ontologies. For consistency in selecting adverse finding descriptions in IVDE and CTD, the user begins by selecting an organ/tissue term, which then restricts the IVDE and MeSH selections to the mapped terms. The user is then directed to select terms that reflect the same or similar findings within IVDE and CTD.

### ToxEvaluator workflow

**Figure 1.**
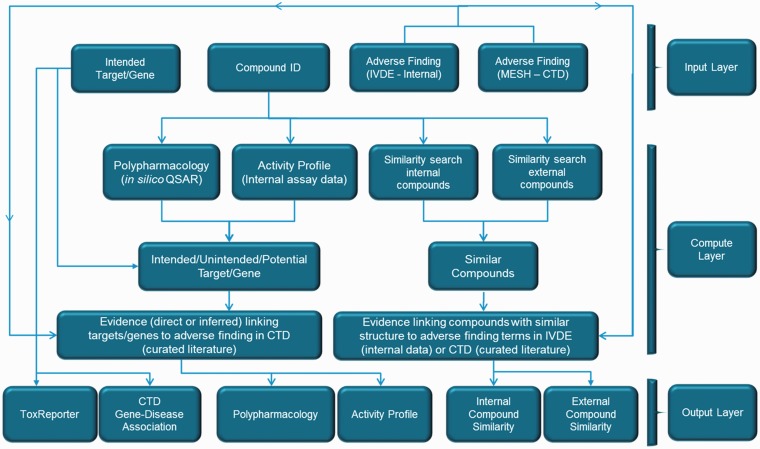
ToxEvaluator Workflow. The ToxEvaluator workflow comprised an input layer, a compute layer and an output layer. The input layer allows the selection of a compound ID, a target and a description of the adverse finding. The compute layer represents the programmatic steps integrated by ToxEvaluator to identify the relationships between the input descriptors that may help support new hypotheses. The output layer displays the compute layer results in six areas that can be defined according to the types of relationships identified.

The workflows integrated in ToxEvaluator are provided in Figure 1. As described above, the input information (top row boxes) consists of the intended target (gene ID), the compound ID and the description of the adverse finding. Computational (similar compound identification, off-target prediction) and data look-up (assay data/secondary target ID) steps based on the input compound form the majority of the initial steps. Secondary targets and similar compounds that cross default thresholds (EC50 < 10 µM and 50% similarity, respectively) are combined with the input target and cross-checked against CTD, IVDE and ToxReporter for linkages to the adverse terms entered.

**Figure 2.**
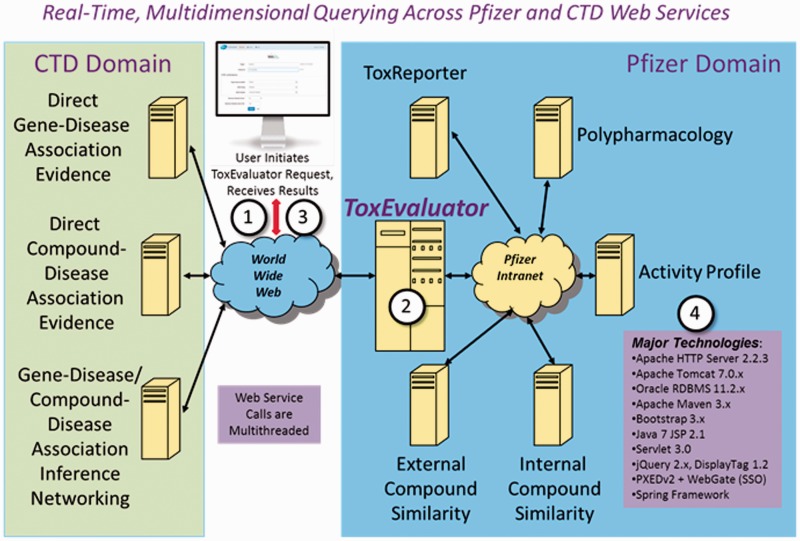
ToxEvaluator Technical Architecture. A ToxEvaluator user (1) initiates a request using the Web-based ToxEvaluator user interface over Pfizer’s virtual/private network. The ToxEvaluator server-side software (2) processes the request, making approximately one dozen multithreaded internal and CTD-based Web service calls, compiling, harmonizing and summarizing the results. A summary report with hyperlinks to more detailed information is then returned (3) to the end user for analysis. Major technologies are summarized (4).

Figure 2 provides an overview of the technical architecture behind ToxEvaluator and a description of the major technologies integrated into the tool. From a software engineering perspective, automating the ToxEvaluator workflows required developers to integrate a highly diverse set of remote information resources. The methods used for accessing these resources were similarly diverse, ranging from Web service- and Common Gateway Interface-based communications, to remote database querying in both MySQL and Oracle environments, to remote process execution. The formats and methods of information exchange were as varied as the resources themselves, including XML, JSON, flat text and direct file-based interchange. When a ToxEvaluator query is executed, over a dozen logically multithreaded, asynchronous remote internal and CTD-based service calls are made to secure, compile and summarize the extracted information. All user queries are saved to an Oracle database management system, enabling users to easily revise and/or rerun queries. The CTD-based services were developed, optimized and intended specifically for Pfizer use. The Java EE 7 computing platform, within the context of Apache/Tomcat and the Spring Framework, was used for software development. These tools collectively provided the functionality and flexibility required to effectively address the disparate demands of ToxEvaluator. Although ToxEvaluator is a Web-based tool, it is accessible only with proper user authentication within Pfizer’s virtual/private network domain.

**Figure 3.**
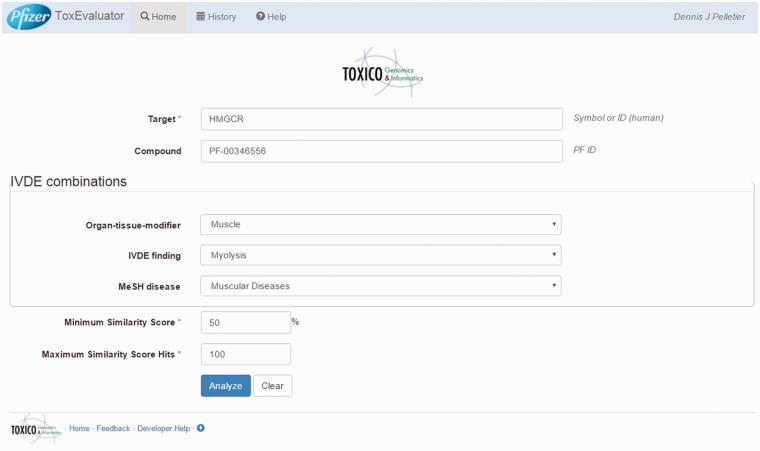
ToxEvaluator input: cerivastatin-myolysis example. A view of the ToxEvaluator input page showing entries specific to the cerivastatin-myolysis example.

**Figure 4.**
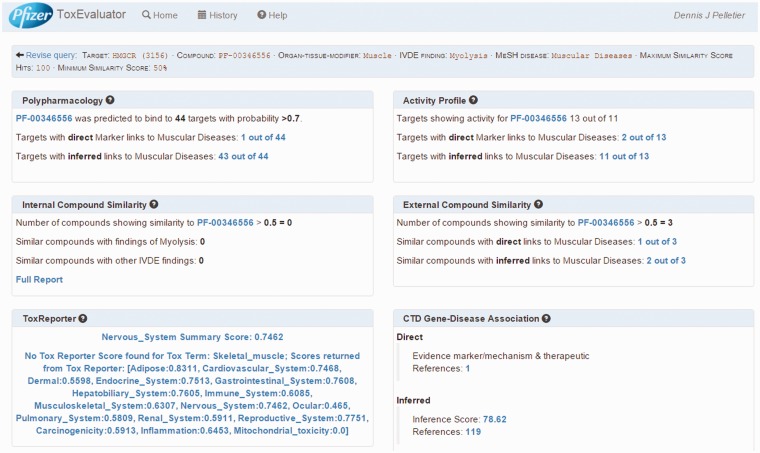
ToxEvaluator output: cerivastatin-myolysis example. A view of the ToxEvaluator output page showing analysis results for the cerivastatin-myolysis example.

The ToxEvaluator logical workflow comprised an input layer, a computation layer and an output layer. The input and output layers are analogous to the ToxEvaluator homepage and report pages, respectively (Figures 3 and 4). The computation layer represents the programmatic steps integrated by ToxEvaluator to identify the links that may support new hypotheses. The output layer contains six result sections grouped according to the types of links they are capable of generating (e.g. through a secondary pharmacology target that was identified via Polypharmacology).

## ToxEvaluator output

### Primary pharmacology output

**Figure 5.**
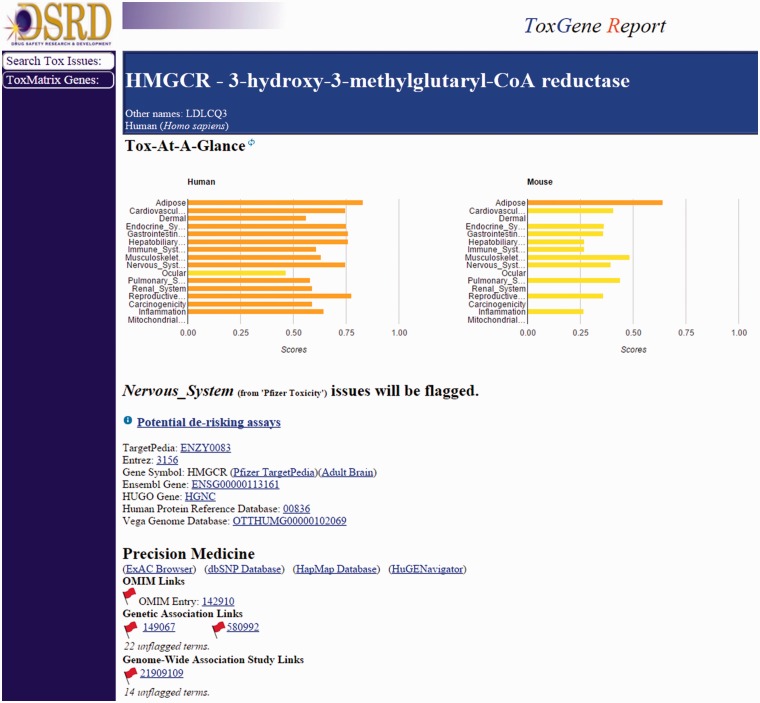
ToxReporter summary page for the HMGCR target. ToxReporter summary page for the input target (HMGCR) showing Tox scores for high-level target organ and mechanism areas across mouse and humans. Flags are visible for those data sources where potential links to the adverse finding of interest have been identified.

Two separate workflows, one internal (ToxReporter) and one external (CTD), focus on the potential role of the primary target in the adverse finding of interest. The CTD Gene–Disease Association report will return any direct or indirect links found between the input target and the adverse finding of interest that have been curated in the CTD from published literature. ToxReporter searches and returns relationships between an input target (i.e. gene) and an adverse finding from various data sources including the OMIM and MGI databases, primary target tissue expression, Gene Ontologies and the literature (PubMed), using the input MeSH term. ToxReporter provides a summary score metric reflecting the number of links found between the target and the safety lexicon term, relative to all other targets. A summary view of ToxReporter output can be seen in Figure 5.

### Structural similarity output

The External Compound Similarity report identifies public-domain compounds with comparable structures and searches for associations to the input finding. Similarly, the Internal Compound Similarity report identifies proprietary compounds with similar structures to the input compound, and associates these compounds with instances where findings identical to the one entered were observed in prior toxicology studies. This report will also alert the user to structurally similar proprietary compounds where any adverse findings were observed in prior toxicology studies. Although we cannot include an example figure of this section as it contains proprietary data, we can report that the data fields include the compound identifier of the similar compound, the similarity score, the study findings, the study title and identifier, and preclinical species used in the study. Live links to the full study report are available as well as in-depth information on the similar compound from internal databases.

### Secondary pharmacology output

The Polypharmacology report identifies computationally predicted off-targets of the compound, and determines whether these off-targets can be linked to the adverse finding via CTD gene–disease relationships. Similarly, the Activity Profile report identifies known (via *in vitro* assay data) unintended targets of the compound, and determines whether these targets can be linked to the adverse finding via CTD gene–disease relationships.

## ToxEvaluator example

### Case study of Cerivastatin-associated myopathy

ToxEvaluator has been used in many internal project-based issue-management initiatives since being developed, often resulting in the rapid identification of compelling hypotheses and informatics and/or experimental follow up. Because its use to-date has been in support of ongoing proprietary programs, we are unable to provide detailed examples of its use at Pfizer. Instead, we include an example using ToxEvaluator to investigate the observation of myolysis with the HMG CoA reductase (HMGCR)-targeting drug cerivastatin to illustrate its utility.

In this example, the user begins on the ToxEvaluator input page (Figure 3) by entering the known drug target, HMGCR, the internal compound identifier for cerivastatin (PF-00346556), and the adverse terms reflecting myolysis in the IVDE (myolysis) and CTD (muscular diseases) databases. Default values for compound similarity (50%) and maximum similarity score hits (100) are left unchanged and the user selects ‘Analyze’.

ToxEvaluator performs the workflows in Figure 1 and returns the output page as seen in Figure 4. ToxEvaluator output is organized according to the underlying connections being made (see ‘ToxEvaluator workflow’ section for details). For example, the Activity Profile section (Figure 4, upper right) reflects findings based on known pharmacological targets of cerivastatin from our proprietary database that have been linked to the input term ‘muscular diseases’ by one or more CTD gene:disease relationships.

### Primary pharmacology output

**Figure 6.**
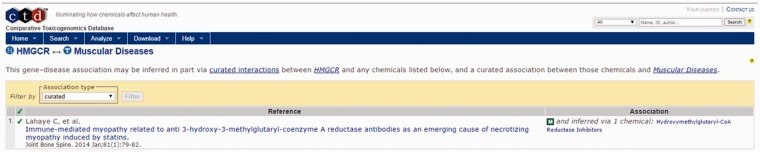
CTD HMGCR—Muscular Diseases reference. CTD target–disease view showing curated article supporting a direct relationship between HMGCR and muscle diseases.

For this example, we focus on four areas of the output page. First is the identification of a direct association between HMGCR, the target of cerivastatin and muscular diseases in the CTD Gene–Disease Association output (Figure 4, bottom right). By selecting the references link in the Direct association section, the user is directed to the CTD Gene–Disease page for HMGCR–Muscular Diseases (Figure 6). The supporting reference there (18) reports a connection between anti-HMGCR antibodies and an immune-mediated form of myopathy in a patient following exposure to statin therapy. This connection might support the hypothesis that an immune-mediated mechanism due to anti-HMGCR antibodies could be contributing to the incidence of myopathy being investigated by the user, a hypothesis that could be tested for supporting experimental evidence.

### External compound similarity output

**Figure 7.**
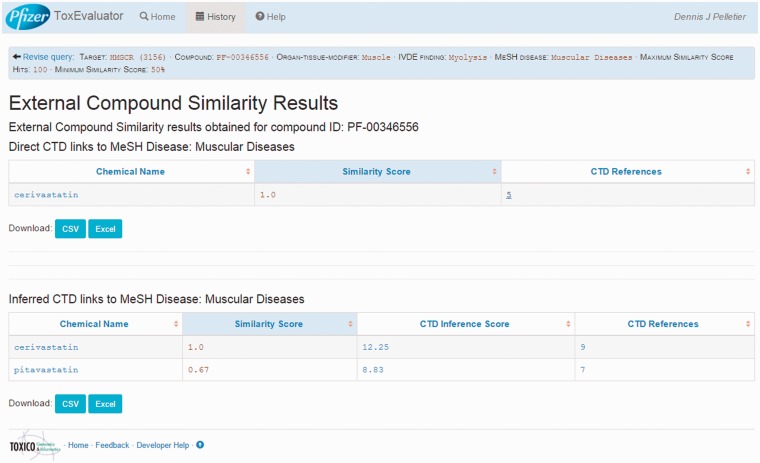
External compound similarity results. External compound similarity results view showing public-domain compounds with similarity to cerivastatin (including cerivastatin) with direct and indirect links curated by CTD to the input term.

**Figure 8.**
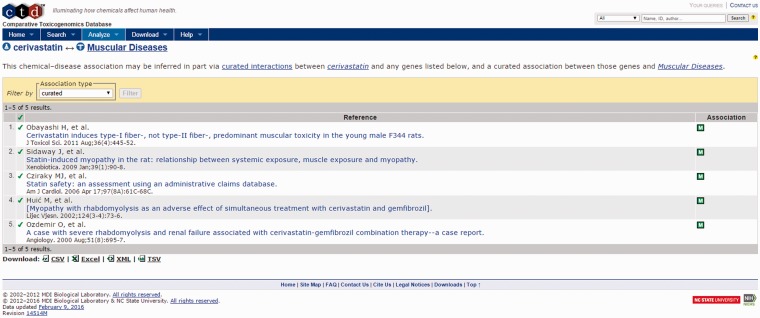
CTD compound–disease view showing curated articles that support a direct relationship between cerivastatin and muscle diseases.

A second area of focus is the identification in the External Compound Similarity output (Figure 4, middle right) of three public-domain compounds with structural similarity to cerivastatin, one of which has a directly curated relationship to the MeSH term ‘Muscular Diseases’. The user may then select the available link to open the External Compound Similarity Results page to investigate this potential lead (Figure 7). In viewing this page, the user sees that the compound of similar structure is cerivastatin itself. Selecting the link under the CTD References area, ToxEvaluator opens the CTD cerivastatin–Muscular Diseases references page (Figure 8), providing the user with a selection of publications supporting the relationship between cerivastatin and this adverse finding (19, 20). This connection, while less useful in terms of building causal mechanistic hypotheses for cerivastatin-associated myolysis, is important in that it demonstrates how ToxEvaluator can also identify known linkages between molecules and adverse findings.

### Polypharmacology output

**Figure 9.**
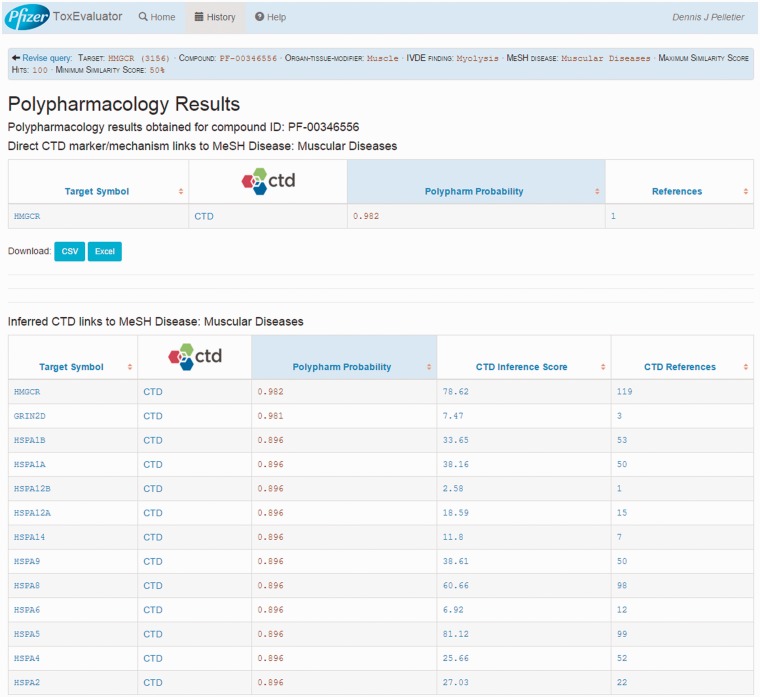
A view of the Polypharmacology results page showing the identification of a predicted high probability target (HMGCR) that has been directly linked to myolysis by CTD.

The Polypharmacology section of the output (Figure 4, upper left) reports the identification of 44 targets that cerivastatin is predicted to interact with and that CTD has linked to muscular diseases. Selecting this link opens the Polypharmacology Results page (Figure 9) where the user is presented with the direct (HMGCR) and indirectly linked predicted targets. Reference links are available for each predicted target for evaluation of supporting evidence by the user and the selection of compelling potential targets for follow-up testing.

### Secondary pharmacology output

The links in the Activity Profile area on the output page are of particular interest for this example. As mentioned previously, the Activity Profile workflow attempts to identify links between known unintended targets (i.e. secondary pharmacology) and the adverse finding of interest. ToxEvaluator does this by identifying targets from a proprietary database where the input compound (e.g. cerivastatin) has demonstrated an IC50 or EC50 value of <10 µM and cross checking these targets at CTD for relationships to the input MeSH term.

**Figure 10.**
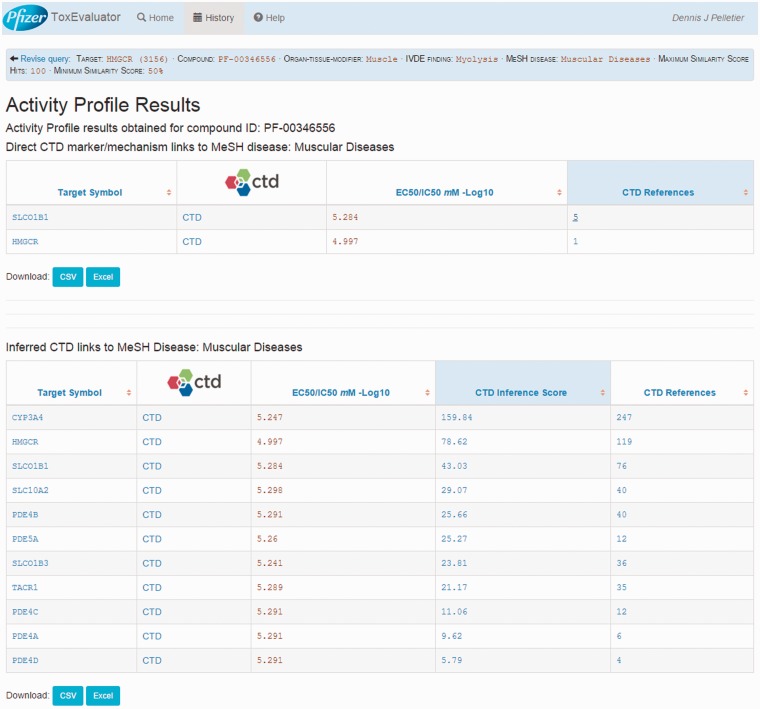
Activity Profile results view showing known targets of cerivastatin from a proprietary database with direct and indirect links curated by CTD to the input term.

**Figure 11.**
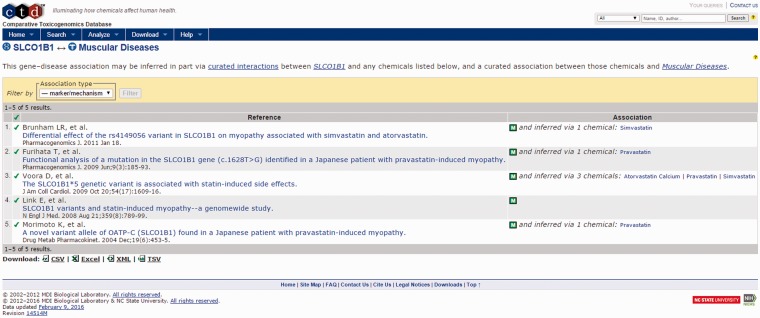
CTD gene–disease view showing curated articles with a direct relationship between an unintended target of cerivastatin (SLCO1B1) and the input term. Together, these direct (via cerivastatin) and indirect (via SLCO1B1) linkages confirm that cerivastatin is associated with myopathy, and suggest that SLCO1B1 inhibition may be causally involved.

For this example, the Activity Profile output reported that 13 secondary targets have been identified for cerivastatin, and that a direct link to muscular diseases was found for two of the targets. Targets with a direct linkage to the disease term have supporting literature that clearly describes a mechanism- or treatment-based relationship between them and are thus connections of primary interest. In cases where targets with direct linkages to the disease term are not identified, targets with indirect linkages could be investigated. Selecting the link available, the user opens the Activity Profile Results page (Figure 10), which identifies the direct-linked known-pharmacology targets associated with muscular diseases as HMGCR (discussed above) and solute carrier organic anion transporter family member 1B1 (SLCO1B1). Selecting the link for SLCO1B1 under the CTD References area, ToxEvaluator opens the SLCO1B1–Muscular Diseases references page at CTD (Figure 11), providing the user with publications supporting the relationship between SLCO1B1 and muscular diseases (21, 22).

SLCO1B1 is a liver-specific organic anion transporter. Functionally, it is involved in the uptake of a number of endogenous (e.g. bilirubin) and small-molecule drugs from the blood into the hepatocytes of the liver. Its identification as a secondary target of cerivastatin by ToxEvaluator and linkage to muscular diseases by CTD may lead the user to derive a hypothesis that inhibition of SLCO1B1 by cerivastatin may inhibit its own clearance from the blood, resulting in elevated serum drug levels, a finding that has been associated with statin-associated myolysis (23).

## Discussion

Serious preclinical toxicology study findings can delay or halt the development of potential new medicines. They can also lead to substantial investigative efforts of the underlying mechanism(s) in order to understand the relevance of the risk to patients or to help avoid the adverse finding for follow-on molecules of the class. Due to the nature of the drug discovery and development processes, the window of opportunity to perform this investigative work is limited, and tools capable of making this work more efficient can have a significant impact on the overall success of the effort. ToxEvaluator was developed as an *in silico* tool with utility to rapidly integrate relevant internal and external data capable of supporting the generation of mechanistic toxicology hypotheses. To accomplish this, it was designed to automate workflows used by investigative toxicologists attempting to understand why a particular adverse finding has been observed.

The software design for ToxEvaluator began with the creation of wireframe diagrams of each workflow to be automated. In this process we captured each step in a workflow from input to output, identifying tools and databases available for each workflow. In some cases, existing workflow tools were leveraged and integrated into ToxEvaluator without modification; in other cases, existing applications had to be modified and/or optimized to accommodate the content and real-time performance demands of ToxEvaluator. New applications were written to allow access to data that was required but not previously remotely accessible. This wireframing process served a number of useful purposes, including providing a description of the workflow with details required for the software development, and an understanding of the disparate formats of the underlying tools and databases required to be integrated. As a result, many of the decisions that went into selecting a particular software tool were made to accommodate the existing and/or new formats and allow their integration within the application. Changes were made to the wireframe diagrams throughout the design and development lifecycle as more was understood about the requirements of ToxEvaluator, and the availability of data sources. The wireframe diagrams proved to be invaluable in providing a simple, common, well-grounded understanding of our design during this highly dynamic process.

Cerivastatin-associated myopathy was used as an example of ToxEvaluator’s utility in uncovering meaningful direct and indirect connections. In this example, a direct relationship was rapidly identified between the primary target of cerivastatin, HMGCR and myopathy in a patient that had been exposed to statin therapy. The identification of anti-HMGCR antibodies in the patient’s blood supported the authors’ assertion that the myopathy is the result of an immune-mediated mechanism (18). Importantly, for the toxicologist using ToxEvaluator, the discovery of this relationship can form the basis to suspect a similar mechanism behind the myopathy under investigation, one that could be tested experimentally for supporting evidence. The relationship between cerivastatin and SLCO1B1 (off-target) that was uncovered in an internal database provided the link to the direct connection between SLCO1B1 and myopathy curated within CTD. Taken together these relationships support a hypothesis that inhibition of SLCO1B1 by cerivastatin could be leading to myopathy. A similar-external-compound (e.g. cerivastatin) to muscle disease connection confirmed the association.

This example illustrates how ToxEvaluator can rapidly, comprehensively and consistently perform investigative toxicology workflows and present the user with relationships among primary sources of information that can form the basis for plausible and testable hypotheses. The automated incorporation of orthogonal data such as structurally similar compounds and unintended pharmacology that can form the basis of mechanistic links to adverse findings is a distinguishing capability of ToxEvaluator. Clearly, it would be impractical for toxicologists to manually render the unified, coherent content provided by ToxEvaluator on a consistent basis, given the volume, complexity, and dynamic nature of an underlying dataset that is only accessible across such a broad and diverse spectrum of resources, platforms and tools. Moreover, although it is conceivable that a skilled investigative toxicologist may have eventually manually uncovered some or perhaps all of these threads of evidence, ToxEvaluator retrieves these findings for the user in seconds. Additionally, by automating these workflows, queries can be rerun easily over time as databases are updated, something that, again, would be impractical using a manual approach.

Although ToxEvaluator represents an important step forward for those engaged in investigative toxicology work, it does have limitations. One of these is the relatively narrow focus of the tools and databases integrated in the current version. To address this limitation, a future version of ToxEvaluator could integrate tools or databases from among the many other publicly available resources (e.g. NCBI, EMBL, etc.). A second limitation for ToxEvaluator is access, which is restricted to internal company use due to the proprietary nature of many of the integrated tools and data. However, our intention in describing the rationale behind this tool, its design and development, and a case study is to stimulate further development of the concept and the availability of similar tools in the public domain. Notably, CTD’s rich, publicly available content could be readily leveraged by any group interested in developing such a tool.

ToxEvaluator represents an evolution in the area of *in silico* tools and databases as applied to toxicology by enabling novel mechanistic insights through the integration of existing resources. As more and newer sources of data are generated and utilized successfully in defined workflows, the likelihood for synergistic gains through their integration to other databases increases. Although this version of ToxEvaluator has automated several commonly used first-order relationships (e.g. known target to disease term), there are additional workflows that could be incorporated in future versions to expand the exploratory workflow options. Examples include the automation and integration of potential secondary target identification methods such as significant sequence identity overlap with the input target, protein domain structural similarity (e.g. for kinase targets), or chemical-genomics approaches (i.e. targets identified via shared active compounds).

## Supplementary Material

Supplementary Data
