# Supplementary material for: ToxEvaluator: an integrated computational platform to aid the interpretation of toxicology study-related findings
Source: Database (Oxford). 2016 May 8;2016:baw062. doi: 10.1093/database/baw062 (PMC4860628; doi:10.1093/database/baw062)
Supplement: Supplementary Data [file supp_2016_baw062_index.html]

Supplementary Data 

# ToxEvaluator: an integrated computational platform to aid the interpretation of toxicology study-related findings

## Supplementary Data

files

- Supplementary Data - xlsx file
